# Multi-Omics and Informatics Analysis of FFPE Tissues Derived from Melanoma Patients with Long/Short Responses to Anti-PD1 Therapy Reveals Pathways of Response

**DOI:** 10.3390/cancers12123515

**Published:** 2020-11-26

**Authors:** Saurabh K. Garg, Eric A. Welsh, Bin Fang, Yuliana I. Hernandez, Trevor Rose, Jhanelle Gray, John M. Koomen, Anders Berglund, James J. Mulé, Joseph Markowitz

**Affiliations:** 1 Department of Cutaneous Oncology, H. Lee Moffitt Cancer Center and Research Institute, Tampa, FL 33612, USA; Saurabh.garg@moffitt.org (S.K.G.); yuliana.hernandez@moffitt.org (Y.I.H.); 2 Biostatistics and Bioinformatics Shared Resource, H. Lee Moffitt Cancer Center and Research Institute, Tampa, FL 33612, USA; eric.welsh@moffitt.org; 3 Proteomics & Metabolomics Core, H. Lee Moffitt Cancer Center and Research Institute, Tampa, FL 33612, USA; bin.fang@moffitt.org (B.F.); John.Koomen@moffitt.org (J.M.K.); 4 Department of Radiology, H. Lee Moffitt Cancer Center and Research Institute, Tampa, FL 33612, USA; trevor.rose@moffitt.org; 5 Department of Oncologic Sciences, University of South Florida Health Morsani College of Medicine, Tampa, FL 33620, USA; Jhanelle.gray@moffitt.org (J.G.); anders.berglund@moffitt.org (A.B.); james.mule@moffitt.org (J.J.M.); 6 Department of Thoracic Oncology, H. Lee Moffitt Cancer Center and Research Institute, Tampa, FL 33612, USA; 7 Department of Biostatistics and Bioinformatics, H. Lee Moffitt Cancer Center and Research Institute, Tampa, FL 33612, USA; 8 Department of Immunology, H. Lee Moffitt Cancer Center and Research Institute, Tampa, FL 33612, USA

**Keywords:** anti-PD-1, melanoma, antigen presentation, informatics, pathways

## Abstract

**Simple Summary:**

Immune based therapies have benefited many melanoma patients, but many patients still do not respond. This study analyzes biospecimens obtained from patients undergoing a type of immune based therapy called anti-PD-1 to understand mechanisms of response and resistance to this treatment. The operational definition of good response utilized in this investigation permitted us to examine the biochemical pathways that are facilitating anti-PD-1 responses independent of prior therapies received by patients. Currently, there are no clinically available tests to reliably test for the outcome of patients treated with anti-PD-1 therapy. The purpose of this study was to facilitate the development of prospective biomarker-directed trials to guide therapy, as even though the side effect profile is favorable for anti-PD-1 therapy, some patients do not respond to therapy with significant toxicity. Each patient may require testing for the pathways upregulated in the tumor to predict optimal benefit to anti-PD-1 treatment.

**Abstract:**

Anti-PD-1 based immune therapies are thought to be dependent on antigen processing and presentation mechanisms. To characterize the immune-dependent mechanisms that predispose stage III/IV melanoma patients to respond to anti-PD-1 therapies, we performed a multi-omics study consisting of expression proteomics and targeted immune-oncology-based mRNA sequencing. Formalin-fixed paraffin-embedded tissue samples were obtained from stage III/IV patients with melanoma prior to anti-PD-1 therapy. The patients were first stratified into poor and good responders based on whether their tumors had or had not progressed while on anti-PD-1 therapy for 1 year. We identified 263 protein/gene candidates that displayed differential expression, of which 223 were identified via proteomics and 40 via targeted-mRNA analyses. The downstream analyses of expression profiles using MetaCore software demonstrated an enrichment of immune system pathways involved in antigen processing/presentation and cytokine production/signaling. Pathway analyses showed interferon (IFN)-γ-mediated signaling via NF-κB and JAK/STAT pathways to affect immune processes in a cell-specific manner and to interact with the inducible nitric oxide synthase. We review these findings within the context of available literature on the efficacy of anti-PD-1 therapy. The comparison of good and poor responders, using efficacy of PD-1-based therapy at 1 year, elucidated the role of antigen presentation in mediating response or resistance to anti-PD-1 blockade.

## 1. Introduction

In 2020, 100,350 new cases of melanoma and 6,850 melanoma-related deaths are projected in the United States [1]. The cancer research community must continue to deepen its understanding of the pathogenic mechanisms of melanoma. The immune system has a role in the development, progression, and effective treatment of melanoma [2]. The coupling of PD (Programmed cell Death protein)-1 on lymphocytes and its ligand PD-L1, which can be expressed on tumor cells and tumor-infiltrating antigen-presenting cells, limits the activity of effector T cells against tumors [3,4,5]. Engagement of PD-1 inhibits the costimulatory signal to the T-cell receptor, resulting in an attenuation of T-cell proliferation and cytokine secretion [6,7,8]. Blockade of this inhibitory immune pathway, using PD-1 neutralizing monoclonal antibodies, has been shown to lead to disease regression, with the response rate to a single anti-PD-1 agent of 40%; however, in combination with an anti-CTLA-4 therapy, response rates to an anti-PD-1 agent increase to 50% to 60%, albeit at the cost of significantly increased toxicity [9,10,11,12,13,14]. Anti-PD-1 monoclonal antibodies are thought to rescue the stimulation process in effector T cells, thereby allowing them to effectively kill melanoma cells. Despite these improved rates of response, many patients do not benefit from these therapies.

Immune-suppressive cytokines, proliferation and recruitment of suppressive immune cells and the metabolic activity of reactive oxygen and nitrogen species can all contribute to dysregulation of the tumor immune microenvironment and lead to evasion of immune checkpoint blockade [15]. In addition, dendritic cell antigen presentation to T cells is known to be dysfunctional in melanoma [16]. The specific process inhibited is the type I and type II interferon (interferon- (IFN-) α and IFN-γ) signaling through the Janus kinase signal transducer and activator of transcription (Jak-STAT) pathway, which supports the ability to enhance tumor recognition by the immune system and facilitate a robust T-cell response [17,18,19].

In the present study, we conducted multi-omics experiments, including proteomics and targeted quantification of mRNA expression performed on clinically available formalin-fixed, paraffin-embedded (FFPE) tissues from a set of melanoma patients who were considered to be good responders or poor responders defined by progression-free survival (PFS) after anti-PD-1 therapy for 1 year. The informatics analysis revealed that proteins involved in antigen presentation and other pathways are implicated in a common response pattern among responders and not dependent on previous types of therapy given to the melanoma patients. This informatics approach can be applied to patient samples in prospective trials analyzing markers of response to anti-PD-1 therapy.

## 2. Results

### 2.1. Patient and Tumor Characteristics

Institutional review board approval was obtained for this study to access biobank-archived FFPE tissue samples collected from patients prior to undergoing anti-PD-1 therapy. These patients had unresectable stage III/IV melanoma, which required systemic treatment. Twenty-five samples were available for the mRNA study, and 19 samples were available for the proteomics study. Clinical characteristics are shown in Table S1 and include the age at start of anti-PD-1 treatment, stage of disease, time to progression, survival on study without disease progression, method of evaluation Response Evaluation Criteria in Solid Tumors (RECIST), the types of prior therapies used for treatment, and the sites of metastatic disease. There is a mix of features, such as prior therapies, in this patient cohort, and sub-analyses were not performed (Table S1). For our analyses, we divided the patients into two groups: poor responders and good responders. We used these definitions, as it was previously shown that patients with fluorodeoxyglucose-negative PET/CT scans at 1 year have prolonged progression-free survival, and melanoma patients may experience pseudo-progression during the first few months of their treatment [20,21]. Poor responders were patients with a progression-free survival <180 days, and good responders were those with a progression-free survival >365 days or stable disease for 6 months after first progression. Patients with pseudo-progression who subsequently responded to therapy for longer than 1 year were also considered to be good responders.

### 2.2. Multi-Omics Studies: Proteomics and Targeted-mRNA Analyses

**Figure 1:**
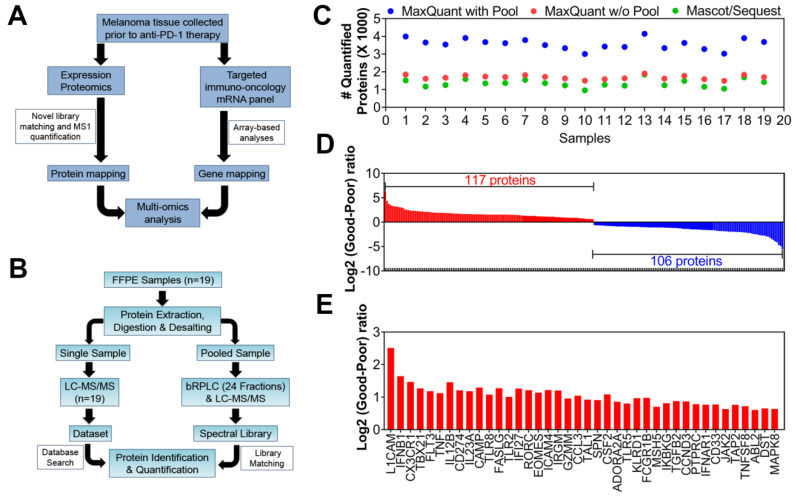
Identification of differentially expressed genes and proteins associated with a favorable response to anti-PD-1 therapy. (**A**) Schematic of the multi-omics pipeline to analyze formalin-fixed, paraffin-embedded (FFPE) samples collected prior to anti-PD-1 therapy in melanoma patients. (**B**) Flow chart describing the library matching process used to significantly increase the identification of unique peptides. (**C**) Scatter plot illustrating the advantage of using library matching to quantify more protein groups by LC–MS/MS across all 19 FFPE samples. (**D**) Two group analyses of proteomics and genomics data were performed on log_2_ transformed and normalized signals. Averages were calculated for each group then subtracted to yield log_2_ ratios. Student’s t-tests (two-sided, unequal variance) and Hellinger distances were calculated to create a score to be used in the informatics pipeline to compare good (progression-free survival (PFS) >1 year) vs poor (PFS <1 year) responders. Two hundred twenty-three differentially expressed proteins (upregulated = blue/downregulated = red) were found in the MaxQuant output based on |log_2_ ratio| ≥ log_2_(1.5-fold change), t-test *p* value < 0.05, and Hellinger distance > 0.25. (**E**) A similar 2 group analysis was then performed on the targeted-mRNA dataset. Forty differentially expressed mRNAs (upregulated = blue) were found based on |log2 ratio| ≥log2(1.5-fold), t-test <0.05, and Hellinger distance >0.25 (Table S3).

We performed multi-omics analyses to examine the protein networks and disease pathways that are essential for a favorable response to anti-PD-1 immune checkpoint blockade therapy. We used biobank-stored FFPE tissue samples from 27 patients with stage III/IV melanoma prior to undergoing anti-PD-1 therapy (Figure 1A, Table S2). Liquid chromatography–mass spectrometry (LC–MS/MS) peptide sequencing and quantification of the individual samples (*n* = 19) were combined with spectral library matching from a pooled sample that was fractionated prior to LC–MS/MS to identify additional proteins in the FFPE tissue samples that would not be routinely sampled in a single LC–MS/MS experiment to increase the depth of the proteome for downstream analysis (Figure 1B). Spectral matching against the data generated from the fractionated pooled sample increased the efficiency of MaxQuant [22] analyses of protein group identification by approximately two-fold across all 19 analyzed FFPE samples (Figure 1C), because peptides that lacked sequence identification in an individual analysis could be matched by accurate mass measurement and retention time to identified peptides in the spectral library or other samples. These 19 FFPE samples were categorized as poor responders (*n* = 10) or good responders (*n* = 9) to perform differential enrichment of protein candidates of proteomics analyses between these two categories. All analyses were performed on the log_2_ transformed and normalized signals [23]. We identified 223 differentially expressed proteins (out of #4958 total) between the two categories utilizing the scoring system (Figure 1D). Of these proteins, 117 were consistently higher in patients with good outcomes, while 106 were consistently expressed at higher levels in patients with poor outcomes (Table S3).

To examine the upregulated expression of genes involved in inflammatory responses to melanoma, a parallel, complementary immuno-oncology-targeted mRNA panel (HTG Molecular Diagnostics, Inc., Tucson AZ, USA; www.htgmolecular.com/assays/io) was used. Twenty-five FFPE samples were submitted to HTG for analysis using the HTG EdgeSeq immuno-oncology assay. As in the proteomic analyses, the FFPE tissue samples were divided into two categories: poor responders (*n* = 11) and good responders (*n* = 14). Downstream expression analyses and 40 upregulated transcripts were identified based on log_2_ ratio ≥ log_2_(1.5-fold change), t test <0.05, and Hellinger distance > 0.25 (Figure 1E; Table S4).

### 2.3. Network Analyses of Differentially Expressed Candidates

#### 2.3.1. Network Analyses of Differentially Expressed Genes and Proteins: Multi-Omics Datasets Analyzed Individually for Processes and Pathways Enriched in Tissues Samples from Good Responders to Anti-PD-1 Therapy

The MetaCore software (Clarivate Analytics, Philadelphia, PA, USA) was used for enrichment analysis, which is the process of identifying gene ontology (GO) terms identified in the datasets at frequencies higher than expected at random. Both publicly available molecular functions and biological processes were analyzed to produce GO terms. Privately curated terms by MetaCore were then used to illustrate the pathway maps (general preconfigured pathways with cellular locations, such as immune response (i.e., the induction of the antigen presentation machinery by IFN-gamma)) and prebuilt process networks (i.e., the study of the interactions between targets that is generally thought of as pathway analysis). The pathway map and process network GO terms are similar to those of biological process/molecular function GO terms that are available from public databases. Using the information generated, networks were built consisting of biological pathways. MetaCore has five different types of network building algorithms: “analyze network”, “analyze network for receptors”, “analyze transcription factors”, “analyze transcription regulation”, and “analyze direct interactions”. MetaCore uses direct interactions to identify clusters of directly interconnected objects from the seed node (the candidate in the dataset) and can be utilized to generate pathways with direct and indirect connections as well as the representation of publicly available GO terms in the pathway generated in the analysis.

**Figure 2:**
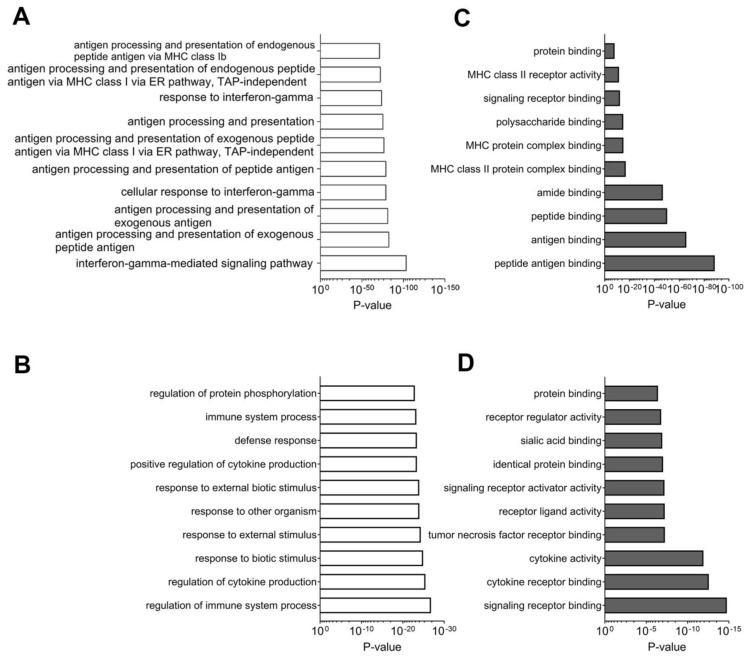
Public gene ontology analysis of candidate genes of proteomics and targeted-mRNA analysis. (**A**,**B**) Analysis for public gene ontology (GO): biological processes. (**A**) Proteomics dataset. (**B**) Targeted-mRNA dataset. (**C**,**D**) Analysis for public GO: molecular function (**C**) proteomics dataset (**D**) targeted-mRNA dataset.

The top 10 GO terms for enriched biological processes identified in proteomics and targeted-mRNA datasets are listed with corresponding *p* values in Figure 2. Most biological processes were associated with immune response and inflammatory signaling. The interferon-γ-mediated signaling pathway and antigen processing and presentation of exogenous peptide antigens were the two most significant processes (Figure 2A; *p* = 1.41 × 10^−104^, 1.12 × 10^−83^). Similarly, targeted-mRNA data analyses showed enrichment of regulation of immune system processes and regulation of cytokine production to be the top GO biological processes (Figure 2B). The molecular functions that were enriched in both datasets consisted of the top GO terms: peptide antigen binding and signaling receptor binding. These terms were used for proteomics and targeted-mRNA sequencing, respectively (Figure 2C,D).

Analyses of multi-omics datasets illustrated enrichment of several tissue and disease-specific inflammatory pathways (Figure S1). The top MetaCore-specific pathway maps enriched in proteomic analyses consist of immune response pathways; these maps were the induction of the antigen presentation machinery by IFN-γ and the maturation and migration of dendritic cells in skin sensitization (Figure S1A). Targeted-mRNA analyses showed enrichment of the role of Toll-like receptor signaling in skin sensitization and attenuation of IFN type I signaling in melanoma cells to be prominent in maps of the enriched pathways, suggesting that the balance between both cells is important for response to anti-PD-1 therapy (Figure S1B). Reiteration of inflammatory response as a dominant factor in the successful outcome of anti-PD-1 therapy was also evident in these networks. The proteomic analyses showed the immune response, with antigen presentation as the most enriched biological process (Figure S1C). Targeted-mRNA analyses were also consistent in the importance of the immune response; T helper cell differentiation was the most enriched biological process in the genomic data (Figure S1D). Analyses of both the datasets suggest that antigen presentation and associated T-cell mediated adaptive responses are important for a response to anti-PD-1 therapy.

#### 2.3.2. Composite Analyses of Multi-Omics Data (Proteomics Combined with Targeted Transcriptomics) Showed the Importance of Antigen Presentation

**Figure 3:**
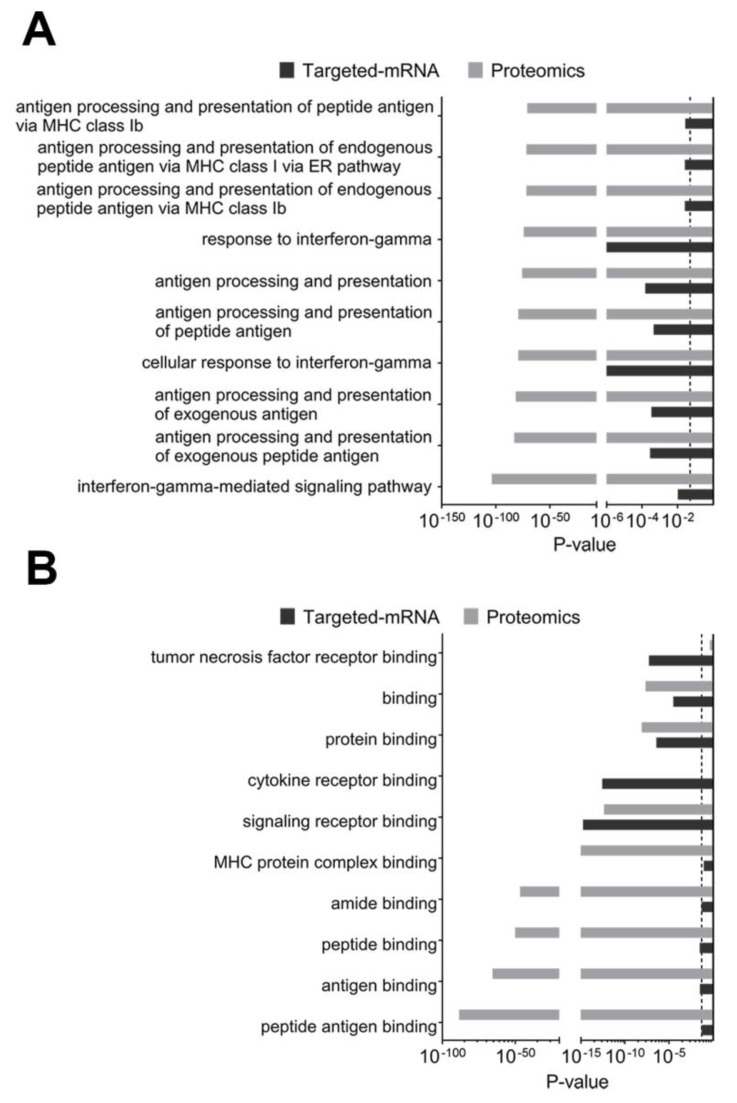
Composite enrichment analysis of proteomics and targeted-mRNA for public gene ontology. (**A**) Analysis for public GO: biological processes. (**B**) Analysis for public GO: molecular function. Dotted line represents *p*-value cutoff of 0.05.

We next performed a GO identification of the most significantly enriched biological processes identified in the cumulative proteogenomic analysis. A public ontology search of biological processes of both proteomic and targeted-mRNA datasets showed the IFN-γ-mediated signaling pathway as well as antigen processing and presentation pathways to be in the top 10 GO terms (Figure 3A). The GO terms that were analyzed for molecular function also complemented the above findings; the top 10 terms included peptide antigen binding and signaling receptor binding (Figure 3B).

Interestingly, MetaCore-specific GO analyses of composite datasets identified the role of the induction of antigen presentation machinery by IFN-γ to be visualized by both pathway maps and network processes (Figure S2).

### 2.4. NETWORK Analyses Illustrates Players of Antigen Presentation Pathways

**Figure 4:**
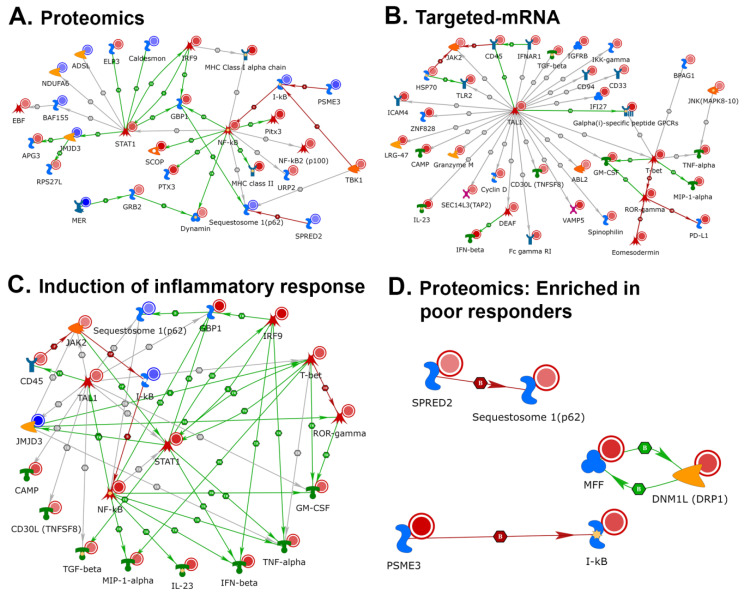
Network analysis of multi-omics data by MetaCore network building tool. (**A**) Proteomics data were analyzed for direct interactions among both upregulated and downregulated seed nodes. (**B**) Targeted-mRNA data were analyzed for direct interactions among upregulated RNA seed nodes. (**C**) Probable signaling cascade to invoke the proinflammatory cytokine response. Network was derived from direct interaction between members with ≥3 connections of both datasets along with candidate cytokines. (**D**) Direct interactions among targets that were upregulated in poor responders.

To characterize the enriched subnetworks, receptors/ligands, and transcription factors (TFs), we used the second step of knowledge-based curation in MetaCore. Proteomics and targeted-mRNA datasets were analyzed using the direct interaction algorithm to create a network consisting of only the seed nodes and their direct interactions. This analysis offered a starting point to identify clusters of directly interconnected objects from the seed node list. The direct interactions between proteomic candidates showed a cluster of 28 proteins that were highlighted by the upregulation of interferon-mediated inflammatory response regulators, such as NF-κB and STAT1, to induce the expression of both MHC I and MHC II molecules in antigen-presenting cells (Figure 4A). A direct interaction analysis of targeted-mRNA datasets showed a cluster of 37 upregulated transcripts. The number of transcripts in this cluster encode for major cytokines that are necessary to pledge, maintain, and manage inflammatory responses; these include TNF-α, GM-CSF, TGF-β, and IFN-β. In the mRNA dataset, this induction is driven by two TFs: TAL1 and T-bet (Figure 4B). We examined the cumulative effect of both datasets on proinflammatory response mechanisms by using the direct interaction algorithm to display the cross talk between the two datasets. A cumulative network analysis was conducted using primary seed nodes with ≥3 connections in individual datasets and all represented cytokines, which illustrates the importance of the proinflammatory response for therapeutic success in using anti-PD-1 therapy (Figure 4C). We also examined direct interaction among proteins that were specifically enriched in poor responders (Figure 4D). This set of proteins only showed connections between Spred2/Squestosome1, MFF-Drp1 and Psme3-IκB.

**Figure 5:**
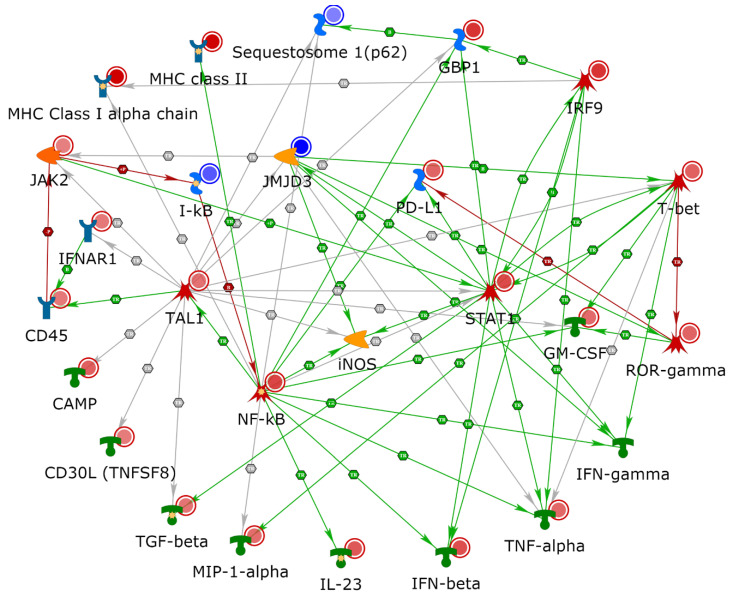
Expansion of direct interactions among cumulative analysis. The cumulative network was derived from direct interaction between members with ≥3 connections of both datasets along with major cytokines. To represent the antigen presentation machinery, MHC class I and class II nodes were added, as was also seen in our data. Given these interactions, interferon (IFN)-γ and iNOS were added as network objects, and both of these objects have direct interactions with nodes found to be regulated in our experimental dataset.

We then performed detailed network building on multi-omics datasets by using four different algorithms: “analyze network”, “analyze network for receptors”, “analyze transcription factors”, and “analyze transcription regulation”. The purpose of conducting these analyses was to identify protein networks with a favorable response to anti-PD-1 therapy. Table S5 describes the top five protein networks of the four different algorithms and the network analyses of proteomic candidates. The “analyze network” algorithm within MetaCore retrieved five protein networks that predominantly consisted of GO processes from public GO biological process databases, such as positive regulation of response to stimuli, antigen processing, presentation by MHC class I and II pathways, and T-cell receptor signaling pathways. The “analyze network for receptors” function showed the top five protein networks that were primarily enriched for GO biological processes of antigen processing and presentation via MHC class I, regulation of protein metabolic process, and regulation of immune response. The “analyze transcription factors” function retrieved the top five protein networks consisting of GO biological processes of antigen processing and presentation via MHC class I and TAP-independent pathways. Our network building analysis on the multi-omics datasets identified seed nodes enriched for networks involved in the top five TFs (SOX17, CREB1, GATA-1, EST1, and TAL1). This analysis yielded GO biological processes, such as cellular metabolic processes, antigen processing, and presentation via MHC class I and the fibroblast growth factor receptor signaling pathway. Similarly, the network analysis of targeted-mRNA candidates is presented in Table S6. The “analyze network function” retrieved the top five networks, which mainly consisted of GO biological processes of the cytokine-mediated signaling pathway, cell surface receptor signaling pathway, and G-protein-coupled receptor signaling pathway. The “analyze network for receptors” function showed enrichment of the top five networks predominantly enriched for GO biological processes of positive regulation of nitrogen compound metabolic processes, positive regulation of cellular metabolic processes, and response to cytokines. The “analyze transcription factors” function retrieved the top five networks consisting of GO biological processes of the cell surface receptor signaling pathway and response to cytokines. The “transcription regulation” analysis illustrated five TFs (AML1 (RUNX1), TAL1, GATA-1, CREB1, and EST1) retrieving GO processes, such as regulation of immune system processes, regulation of cytokine production, and defense responses to other organisms. This analysis suggested that inducible nitric oxide synthase (iNOS) should also have direct interactions in pathways associated with response to PD-1 therapy, and when added to the model, iNOS has multiple direct interactions (Figure 5).

Overall, network building in MetaCore illustrated that both datasets were enriched in proteins and transcripts essential to efficiently process/present tumor-associated antigens and to sustain optimal immune surveillance in response to anti-PD-1 therapy in metastatic melanoma patients. Enrichment analysis implicated the role of cytokine signaling, antigen processing/presentation pathways and T cell-mediated adaptive immune responses to be the major contributors of the represented differences of good and poor responders of anti-PD-1 therapy. Moreover, detailed network analysis either using receptor-centric or transcription factor-centric approaches yielded antigen presentation via MHC class I and II pathways to be the central theme that is responsible for the favorable outcome of subsequent anti-PD-1 therapy.

### 2.5. Use of TIMER2.0 to Determine Immune Cell Populations in Patient Specimens

To examine the types of immune cell populations in the tumor microenvironment, we performed TIMER2.0 analysis on the targeted-mRNA dataset. We compared poor and good responders to anti-PD1 therapy. The TIMER algorithm estimated an increase in CD8^+^ tumor infiltrated lymphocytes in anti-PD-1 responsive tumors (Figure S3A). Elevated frequencies of melanoma infiltrating CD8^+^ T cells in good responders corroborates previous studies that have demonstrated that increased cell infiltration is associated with beneficial clinical outcomes of patients in response to anti-PD1 therapy [24]. Consistent with accumulation of CD8^+^ T cells, cytotoxicity also increased as estimated by Microenvironment Cell population (MCP)-counter (Figure S3B). In contrast, CD4^+^ T cells showed a reverse trend of decreased frequencies in good responders (*p* = 0.0333), which may be due to the reduced numbers of T regulatory cells in the samples from anti-PD-1 responsive tumors (Figure S3C). In the literature, decreased numbers of Treg in the tumor microenvironment overcome melanoma resistance to anti-PD-1 therapy [25]. The Estimating the Proportion of Immune and Cancer cells (EPIC) analysis showed decreased numbers of endothelial cells in good responders to anti-PD1 therapy (Figure S3D), implicating increased frequency of endothelial cells in pro-angiogenesis and tumor progression [26,27]. Overall, the immune infiltration estimations by TIMER2.0 provided evidence for cell populations forming a tumor microenvironment favorable to anti-PD-1 blockade therapy.

## 3. Discussion

In this study, we utilized parallel proteomics and targeted-mRNA analyses to identify protein and mRNA candidates that were differentially altered in the FFPE samples of patients whose tumors responded well to anti-PD-1 therapy as opposed to tumors that were not responsive. We used 1 year PFS as a benchmark to define a good responder to anti-PD-1 therapy as per RECIST criteria. To analyze proteomics data, we used MaxQuant combined with spectral mapping, which yielded high-resolution quantitative data with high-peptide identification rates, increased mass accuracies and proteome-wide protein quantification [22]. To measure upregulated immune-oncology-related mRNA transcripts, we used an established next-generation sequencing- (NGS)-based HTG EdgeSeq Precision immuno-oncology panel, which is designed to measure the immune response both inside the tumor and in the surrounding microenvironment [28,29]. The pathway analysis was conducted using MetaCore software, an ingenuity-based network analysis tool that has been widely used for analyzing gene-expression profiles to identify protein networks in cancers [30,31,32]. 

Expectedly, the output from proteomics and targeted-mRNA approaches shared common pathways but had limited overlap in the terms of identified candidates. Protein half-life and subcellular localization (secreted vs cellular) play a critical role in its representation in the identified dataset. For example, the targeted-mRNA illustrated six elevated cytokines in good responders (GM-CSF, TNF-α, TGF-β, IFN-β, IL-23, and MIP-1-α) that were not represented in the proteomics data due to shorter protein half-life or due to the fact that the specimens were derived from FFPE tissues (cytokines secreted into the extracellular space and not detected in FFPE samples). The analysis of cytokine pathways based on the threshold of ≥3 connections per candidate (Figure 4C) revealed significant connectivity between the proteomics and targeted-mRNA candidates. The transcription factors, NF-kB, STAT1, IRF9, found in the proteomics analysis induce the expression of all six cytokines identified by the targeted-mRNA approach (Figure 4C). Furthermore, both the datasets also shared significantly enriched GO processes such as “cellular response to IFN-γ” and “antigen processing and presentation”, attesting to their significant biological overlap without sharing the same genes for encoded proteins/transcripts.

In our enrichment analyses, we demonstrated an association between favorable anti-PD-1 treatment and induction of antigen presentation machinery by IFN-γ as well as maturation and migration of DC pathways. Moreover, when IFN-γ was added to the direct interaction analyses of multi-omics data, we observed enrichment of elevated NF-κB and JAK/STAT pathways to be stimulated by increased IFN-γ, which in turn increased NF-κB and STAT1 (Figure 5). This observation is substantiated by the fact that the IFN-γ promoter contains an NF-kB-responsive element and undergoes transcription activation through activated STAT1 [33,34,35]. IFN-γ is mainly secreted by activated lymphocytes, including CD4 and CD8 T cells, γδ T cells, and NK and NKT cells [36,37,38,39]. Furthermore, B cells also produce IFN-γ and have gained attention due to their emerging role in sustained inflammation in response to immunotherapy [40,41,42,43,44,45,46]. Melanoma-associated B cell-rich tertiary lymphoid structures are involved in improved antigen presentation, enhanced cytokine signaling/release of tumor-specific antibodies and are linked to improved prognosis [43,47]. Apart from the induction of antigen presentation of DCs and macrophages by tumor-infiltrating lymphocytes, IFN-γ has been implicated in a number of antitumor effects, such as promotion of inflammatory events, leukocyte activation, anti-proliferation, and anti-angiogenesis [48,49,50,51,52,53]. 

Tumor-infiltrating DCs are a major component of antigen presentation machinery, as they elicit an efficient antitumor response to cytotoxic T lymphocytes by capturing and processing the tumor antigens [54,55,56]. In addition to the role of tumor neoantigen-specific CD8^+^ T cells in cancer elimination through MHC class I [57,58,59,60,61], enhanced antitumor activity requires activation of CD4^+^ T cells via MHC class II in response to immunotherapy [62]. Furthermore, anti-PD-1 effects are dependent on MHC II activities [63]. MHC class I expression is primarily regulated by NF-κB and IFN-regulatory factors in response to IFN-γ stimulation [64]. NF-κB expression also leads to the expression of MHC class II genes in response to inflammatory stimuli of IFN-γ [65,66]. Consistent with induced IFN-γ signaling, our multi-omics analyses showed activation of the NF-kB signaling pathway and MHC class I and MHC class II expression. Examination of direct interactions among proteomics and targeted-mRNA datasets showed NF-κB, STAT1, and IRF9 to be implicated in the activation of MHC class I and II expression via induction of proinflammatory T-cell response, which is initiated through cytokines, as shown in Figure 4C. Notably, detailed network analyses of both datasets (Tables S4 and S5) identified multiple networks that were enriched for processes involved in antigen presentation via MHC class I/II proteins and antigen presentation via STAT-dependent and TAP-independent proteins. This involvement indicated a significant role of induced MHC proteins in priming host immune responses to a plausible, sustained inflammatory state in patients responsive to anti-PD-1 therapy.

Prior to the era of checkpoint blockade, vaccine therapies in melanoma had limited results in the treatment of melanoma patients. However, there is a rich history in understanding the use of adjuvants (e.g., cytokines such as interferon and GM-CSF, mycobacterial protein components, dendritic cells) in vaccine therapy in the treatment of melanoma patients [67]. Recent vaccine studies have attempted with varying degrees of success to turn immune infiltrate poor microenvironments into tumor microenvironments suitable for checkpoint blockade including the use of adjuvants such as Toll-like receptor ligands and STING agonists [68,69]. The timing and dosing of the adjuvant is important for melanoma therapy [67,68,69]. All of these adjuvants are known to upregulate parts of the antigen presentation pathway and therefore the informatics results from a given patient may be useful to personalize the adjuvant utilized in future melanoma studies. 

The biological processes noted above led us to analyze the specific networks. Knowledge-based curation of transcription networks that are accountable for the induction of inflammatory events showed enrichment of networks involving TAL1, SOX17, GATA-1, RUNX1, CREB1, and ETS1. Furthermore, we showed TAL1 to be directly connected to NF-κB signaling when both proteomics and targeted-mRNA datasets were combined for direct interactions (Figure 4C) [70]. TAL1 is an essential transcription factor for normal hematopoiesis [71,72]. Intriguingly, increased TAL-1 activity in T cells increased the lifespan of lymphocytes [73]. Our targeted-mRNA dataset showed increased expression of TAL1 to be primarily involved in immune system processes, as indicated by the direct interaction (Figure 4B) and network analysis algorithms, through transcription regulation (Tables S2 and S3). Notably, TAL1 collaborates with several transcription factors (including GATA-1, RUNX1, and ETS1) involved in hematopoietic reprogramming [71,72,74]. 

Cell-proliferation-promoting cytokines, including TNF-α, GM-CSF, IL-23, MIP-1-α, and CD30L, displayed direct interactions with NF-kB and JAK/STAT signaling pathways, which induced an inflammatory network as shown in Figure 4C. Importantly, anti-proliferative cytokines TGF-β and IFN-β were also induced, thereby indicating the presence of costimulatory signals that uncouple antitumor immunity and autoimmune toxicity induced by anti-PD-1 therapy [75,76,77,78,79].

To predict clinical benefits from immunotherapies, biomarker screening via immunohistochemistry of tumor cells initially focused on PD-L1 expression status. A correlation between high PD-L1 expression and clinical response has been reported in studies on anti-PD-1 treatments [80,81,82]. However, this correlation does not explain the clinical responses observed among patients with low or no PD-L1 expressing tumors, possibly due to sample heterogeneity and limited assay standardization [82,83].

The present study suggests that IFN-γ-induced pathways and subsequent antigen presentation are necessary for a response to anti-PD1 therapy. However, persistent type II interferon signaling via STAT1 also promotes induction of genes necessary for encoding ligands for T cell exhaustion and immune escape such as PD-L1 [84]. The targeted-mRNA data showed upregulation of PD-L1, a type II interferon-induced gene responsible for engaging lymphocytic PD-1 to inhibit T cell effector function. Furthermore, the literature suggests that IFN type I and II signaling with subsequent PD-L1 expression on tumor cells and immune cells (i.e., tumor associated macrophages (TAM)) was a major contributor of tumor immune escape [85]. Therefore, the dichotomy of PD-L1 expressing tumors facilitating immune escape while promoting a response to anti-PD1 blockade is supported by our multi-omics findings, similar to other studies [63,86].

Other metrics of response, including clinical measures, such as eosinophilia and sites of metastatic disease, have also been observed in studies on the anti-PD-1 therapy drug pembrolizumab, but these metrices are not in routine clinical use [87,88]. Nevertheless, PD-L1 levels were upregulated in the targeted-mRNA dataset mostly in response to elevated NF-κB and STAT1 expression (Figure 5). Recently, attempts have also been made to identify resistance signatures (e.g., innate anti-PD-1 resistance) prior to anti-PD-1 therapy [89,90]. Although we can identify subpopulations with response to this therapy based on these studies, prediction of sensitivity or resistance among all anti-PD-1-treated melanoma patients remains a challenge.

There were fewer protein networks in common across all non-responders as compared to the number of proteins responsible for response to anti-PD-1 therapy. As reported in the literature, sequestosome1, MFF, Drp1 and Psme3 are known to be associated with poor prognosis in melanoma and other cancers but these negative factors were found in separate studies whereas we were able to find all of these factors using our informatics approach [91,92,93,94]. 

Initially, CTLA-4 was predicted to condition for efficacy in the priming phase of the immune response whereas PD-1 was thought to be involved in the effector phase [95]. However, there is evidence demonstrating that anti-PD-1 administration prior to anti-CTLA4 administration may be more efficacious [96,97]. BRAF inhibition is known to permit infiltration of T cells and together with MEK inhibition increases antigen presentation in melanoma models [98]. A recent multicenter cohort study incorporating PD-1 based therapy with a BRAF/MEK backbone supports this hypothesis [99]. Recently, the PD-L1 inhibitor was Food and Drug Administration (FDA) approved in combination with the BRAF/MEK inhibitor combination of vemurafenib/cobimetinib [100]. However, as with combination immune therapy, it is not yet entirely clear who would benefit from sequential administration versus upfront combination treatment. There is also controversy over whether chemotherapy preconditions patients for anti-PD-1 success. Dacarbazine may condition for anti-PD-1 success by upregulating MHC-I expression [101,102]. In esophageal and other cancers, carboplatinum/paclitaxel chemotherapy was shown to increase PD-L1 expression [103,104]. Although there are some studies reviewing the potential synergistic effects of chemotherapy with anti-PD-1 [105], there is only one small case series to date suggesting the benefit of combination chemotherapy with PD-1 blockade after anti-PD-1 failure [106]. However, melanoma patients who received anti-PD-1 as a front-line therapy had increased responses compared to those patients who received prior therapies including chemotherapy in the original anti-PD1 trials [107]. Interferon therapy is utilized less frequently these days due to toxicity. This being said, IFN-α/β secreting innate immune cells in close proximity to tumor infiltrating DCs form a pro-inflammatory niche essential for enhancing antigen processing/presentation of MHC class I/II and subsequent priming of effector T cell responses [108]. Consistent with our findings, combinatorial approaches using pembrolizumab/pegylated-IFN have demonstrated promising evidence of clinical efficacy in a subset of advanced melanoma patients [109]. Therefore, the message is that immune-targeted and chemotherapy all modulate the tumor microenvironment and at this point we do not have a rationale strategy to combine these agents in all patients. In all likelihood, the strategy for combination will need to be a personalized approach and the optimization of the treatment regime of these agents is important to maximize patient benefits.

Myeloid-derived suppressor cells (MDSCs) are also a known source of immunosuppression that mediates resistance to anti-PD-1 therapy [96,110]. In humans, the definition of MDSCs includes an immature myeloid phenotype (variation of: CD33^+^, CD11b^+^, and HLADR^low/-^) and the ability to suppress the function of other immune cells (e.g., T cells). The number of MDSCs increase with more advanced stages of melanoma, and this change is associated with poor survival [111]. In addition, these increased MDSC populations are associated with poor anti-PD-1 response in melanoma patients [96]. Production of nitric oxide (NO) by MDSCs leads to the production of reactive nitrogen species, which are chemical entities inside cells derived from NO (e.g., peroxynitrite) that cause nitration in key amino acids, such as tyrosine [112,113]. A mechanism of immune inhibition by MDSC in melanoma was proposed that involves secretion of NO by MDSCs and decreased p-STAT1 signaling in response to IFN signaling [113,114]. NO inhibits antigen presentation from DCs to CD4^+^ T cells in the presence of MDSCs in melanoma cells, and nitrated STAT1 can be found in melanoma specimens [115]. We recently reported that in patients treated with anti-CTLA-4 therapy and higher levels of NO in suppressor and effector immune cells were associated with poor and good prognosis, respectively, thereby suggesting NO signaling to have both a pro- and antitumor role [116]. Given the pathways observed in this study, this dichotomy may also exist in patients who receive anti-PD-1 therapy and warrants further investigation to determine how response to anti-PD-1 is mediated by multiple antigen presentation pathways.

## 4. Materials and Methods

### 4.1. Collection of FFPE Samples and Clinical Information

FFPE samples were obtained under an Institutional Review Board (IRB)-approved protocol (MCC 18583, Advarra) from patients who had received a biopsy within 1 year prior to the start of anti-PD-1 therapy. Twenty-five samples were available for mRNA analysis, and 19 samples were available for proteomic analyses. PFS was calculated with the assistance of scans read via RECIST criteria by a staff radiologist.

### 4.2. Mass Spectrometry Analysis

To overcome the dynamic range in protein amounts from abundant, such as actin, to minute, proteomic workflows, we typically used peptide prefractionation and liquid chromatography–mass spectrometry (i.e., LC–MS/MS) [117]. Even with multiplexing, instrument time and cost remain barriers to the analysis of large cohorts [118]. Furthermore, some clinical samples (such as tissue microarray or laser capture microdissected specimens) have limited quantities of total protein (i.e., 1–2 μg) and are too small for effective prefractionation, [119] such as the ones in this study. Therefore, digests of human FFPE melanoma tissue samples were analyzed using LC–MS/MS without prefractionation, but an alternative strategy can be used to generate additional protein identification and quantification. A pooled sample was created from excess material from each tryptic digest, fractionated with basic pH reversed-phase liquid chromatography, and analyzed with LC–MS/MS. Then, the data analysis strategy includes peptide sequence identification from the individual LC–MS/MS data generated from each patient sample and matched to a library generated from the multiple LC–MS/MS analyses of the fractionated peptides from the pooled sample, which contained an equal mixture of all the samples in the patient cohort (Figure 1). Relative quantitation was significantly enhanced by matching the data to a comprehensive peptide library generated from the analysis of the pool in the same batch of samples. This matching greatly increased the number of peptide identifications, thereby providing more evidence for the measurement of each protein. 

FFPE tissue slices were deparaffinized with xylene, then rehydrated sequentially in 100%, 85%, and 70% ethanol. To assist in antigen retrieval, incubations with aqueous ammonium bicarbonate were performed for 2 h at 80 °C and 1 h at 60 °C, followed by sonication. After measuring the protein concentration, the proteins were reduced, alkylated, and digested with trypsin (enzyme-to-substrate w/w ratio = 1:20), and the peptides were desalted using SepPak C18 cartridges (Hypersep C18, ThermoFisher, Waltham, MA, USA). A pooled peptide library was generated from an LC–MS/MS analysis of 24 fractions concatenated after basic pH reversed-phase liquid chromatography [120]. A Dionex U3000 nanoUPLC coupled to a Q Exactive Plus mass spectrometer (ThermoFisher Waltham, MA, USA) was used to analyze the fractions and each individual FFPE tissue sample. The software packages Mascot and Sequest contained in Proteome Discoverer (Thermo) were used to identify the proteins, and MaxQuant analyses were performed for relative quantification [22,121,122].

### 4.3. Targeted-mRNA Analyses

FFPE samples were run on an HTG EdgeSeq Processor using the HTG EdgeSeq immuno-oncology assay (HTG Molecular Diagnostics, Inc., Tucson AZ, USA; www.htgmolecular.com/assays/io). The next-generation sequencing- (NGS)-based HTG EdgeSeq Precision immuno-oncology panel measures a set of 549 immune-oncology-related genes and was used in the multi-omics experiments.

### 4.4. TIMER2.0 Analysis

Normalized targeted-mRNA data were utilized for estimating the immune cells in the tumor microenvironment using the in silico tool TIMER2.0 (http://timer.cistrome.org/). TIMER2.0 provides immune cell infiltration estimations for user-provided mRNA expression profiles by TIMER, CIBERSORT, quanTIseq, xCell, MCP-counter and EPIC algorithms [123,124,125,126,127,128]. The analysis was performed using settings for human skin cutaneous melanoma (SKCM). The Mann–Whitney test (GraphPad Prism, San Diego, CA, USA) was used to calculate the significant differences of immune infiltration between poor and good responders to anti-PD1 therapy. 

### 4.5. Analysis Considerations

The data generated in the mRNA and proteomic experiments were analyzed. Within a sample derived from a patient, the mRNA panel was normalized by dividing the abundance of each mRNA by the geometric mean of 14 (out of 15) housekeeping genes. One housekeeping gene, *TBP*, was excluded from the analysis due to low signal intensity. A scaling factor of 400,000 was applied because it was the lowest multiple of 100,000 to result in no log_2_ values <1. Protein spectra, quantified using MaxQuant (Max Planck Institute, Martinsried, Germany) [23,129], were normalized using IRON (Iterative Rank-Order Normalization) [23] (iron_generic --proteomics) against the median sample (findmedian --spreadsheet --pearson). Downstream 2-group differential expression analyses were performed (good and poor responders) and filtered using the following cutoffs: log_2_ ratio ≥log_2_ (1.5-fold change), t test <0.05, and Hellinger distance >0.25. For proteomics data, an additional filter excluded rows mapping to bovine proteins or entirely to reverse amino acid sequences. Gene lists were pruned from the proteomic and mRNA experiments to keep only those genes observed in their respective experimental proteomics, mRNA, or combined datasets. This pruning was completed to account for the immune bias of the mRNA targeted panel. Pathway enrichment of differentially expressed genes was performed by applying Fisher’s exact test to the Molecular Signatures Database (MSigDB) gene lists [130,131,132]. Prior to pathway enrichment of differentially expressed gene lists, the MSigDB gene lists were filtered to keep only those genes observed in their respective experimental proteomics, mRNA, or combined datasets. This prefiltering was performed to account for the immune-related bias of the targeted-mRNA panel. Literature interaction networks of differentially expressed genes were generated with MetaCore (Clarivate Analytics, Philadelphia, PA, USA). 

## 5. Conclusions

In summary, multi-omics analyses illustrated the importance of antigen processing and presentation pathways in activating the immune system to initiate and maintain the inflammatory response to anti-PD-1 therapy in metastatic melanoma patients. The operational definition of good response utilized in this investigation permitted us to dissect out the pathways that are facilitating anti-PD-1 responses independent of prior therapies received by patients. Currently, there are no tests to reliably test for the outcome of patients treated with anti-PD-1 therapy. The purpose of this study was to facilitate the development of prospective biomarker-directed trials to guide therapy, as even though the side effect profile is favorable for anti-PD-1 therapy, some patients do not respond to therapy with significant toxicity. Each patient may require testing for the pathways upregulated in the tumor microenvironment.

## Supplementary Materials

The following materials are available online at https://www.mdpi.com/2072-6694/12/12/3515/s1, Figure S1: MetaCore curated gene ontology analysis of candidate genes of proteomics and targeted-mRNA analysis. (A–B) MetaCore GO analysis for curated pathway maps representing complete biochemical pathways or signaling cascades that are enriched in analyzed dataset. (A) Proteomics dataset. (B) Targeted-mRNA dataset. (C–D) Ontology analysis for pre-build networks in MetaCore such as biological processes, toxicity, metabolic processes, disease biomarkers etc. for proteomics dataset. (C) Proteomics dataset. (D) Targeted-mRNA dataset; Figure S2: Composite enrichment analysis of proteomics and targeted-mRNA for MetaCore Gene Ontology. (A) Analysis for MetaCore GO: pathway maps. (B) Analysis for MetaCore GO: networks. Dotted line showing *p*-value cutoff of 0.05; Figure S3: Examination of immune cell population in the clinical specimens. TIMER2.0 was performed on the targeted-mRNA dataset to understand which cells were present in FFPE tissue samples from poor and good responders to anti-PD1 therapy. (A) Violin plot showing significant increase in frequencies of CD8^+^ lymphocytes in good responders to anti-PD1 therapy estimated by TIMER. (B) Violin plot showing significant increase in cytotoxicity score of good responders to anti-PD1 therapy, estimated by MCP-counter. (C) Violin plot showing significant reduction in frequencies of CD4^+^ lymphocytes in good responders to anti-PD1 therapy estimated by TIMER. (D) Violin plot showing significant reduction in frequencies of endothelial cells in good responders to anti-PD1 therapy estimated by EPIC. *p*-values calculated using Mann–Whitney test. **p* ≤0.05; Table S1: Clinical characteristics of FFPE samples; Table S2: Distribution of FFPE samples for multi-omics study; Table S3: Expression proteomics list; Table S4: Targeted-mRNA list; Table S5: Top five network lists analyzed by network building via MetaCore that are enriched in proteomics data; Table S6: Top five network lists analyzed by network building via MetaCore that are enriched in targeted-mRNA data.
