# Supplementary material for: Multi-Omics and Informatics Analysis of FFPE Tissues Derived from Melanoma Patients with Long/Short Responses to Anti-PD1 Therapy Reveals Pathways of Response
Source: Cancers (Basel). 2020 Nov 26;12(12):3515. doi: 10.3390/cancers12123515 (PMC7768436; doi:10.3390/cancers12123515)
Supplement: Supplementary file 1 [file cancers-12-03515-s001.zip › supplemental_material/Suppl_figures_11242020.pdf]

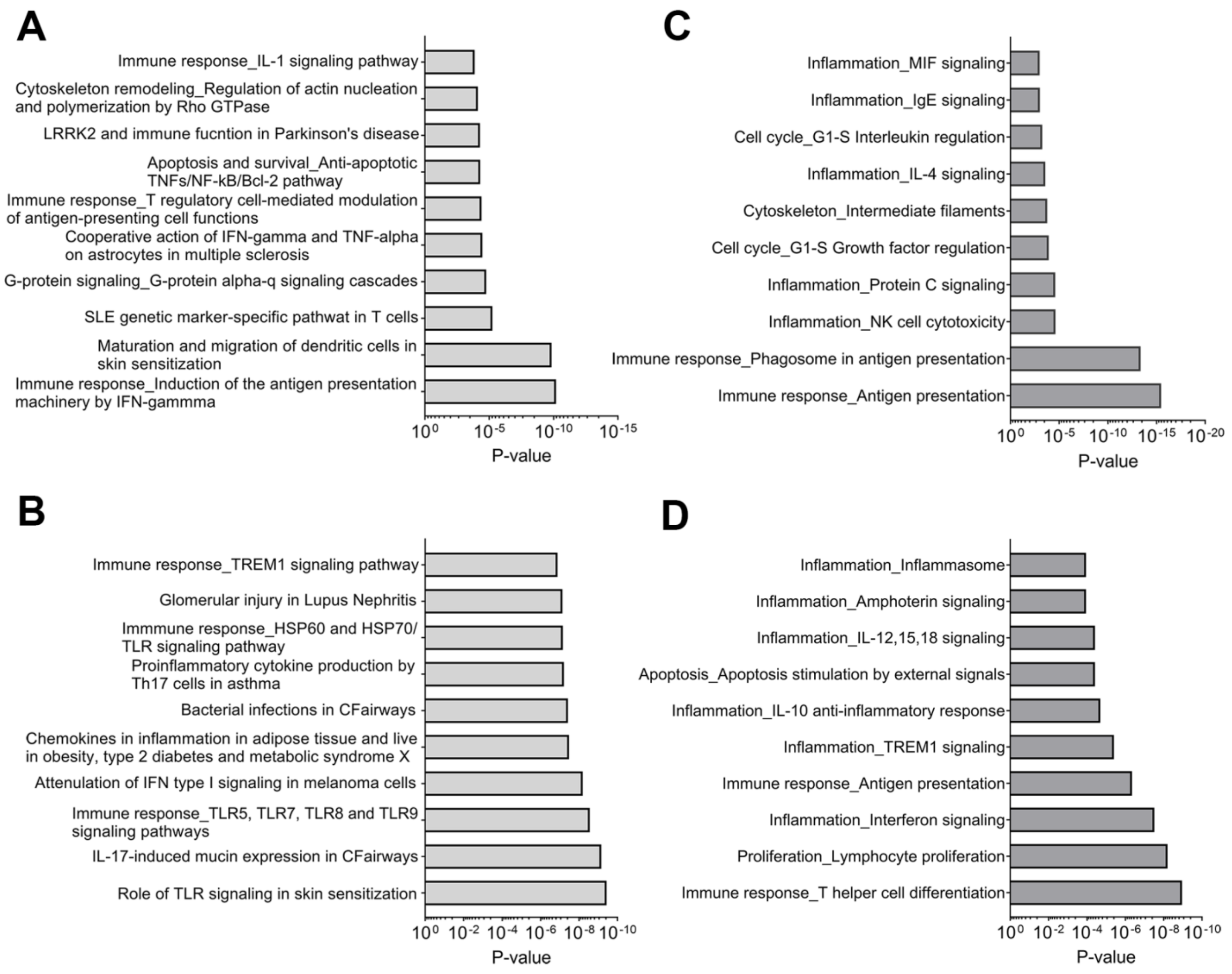

Figure S1: MetaCore curated gene Ontology analysis of candidate genes of Proteomics and targeted-mRNA analysis. (A-B) MetaCoreGO analysis for curated pathway maps representing complete biochemical pathways or signaling cascades that are enriched in analyzed dataset. (A) Proteomics dataset. (B) Targeted-mRNA dataset. (C-D) Ontology analysis for pre-build networks in MetaCoresuch as biological processes, toxicity, metabolic processes, disease biomarkers etc. for proteomics dataset. (C) Proteomics dataset. (D) Targeted-mRNA dataset.

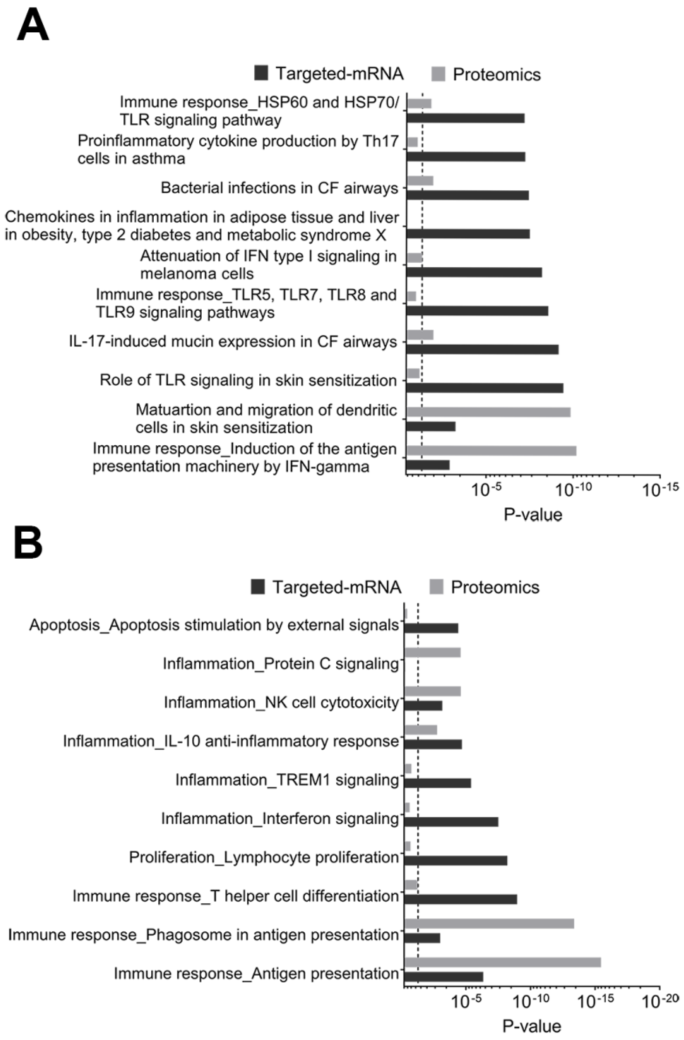

Figure S2: Composite enrichment analysis of proteomics and targeted-mRNA for MetaCore Gene Ontology. (A) Analysis for MetaCore GO: pathway maps (B) Analysis for MetaCore GO: networks. Dotted line showing *p*-value cutoff of 0.05.

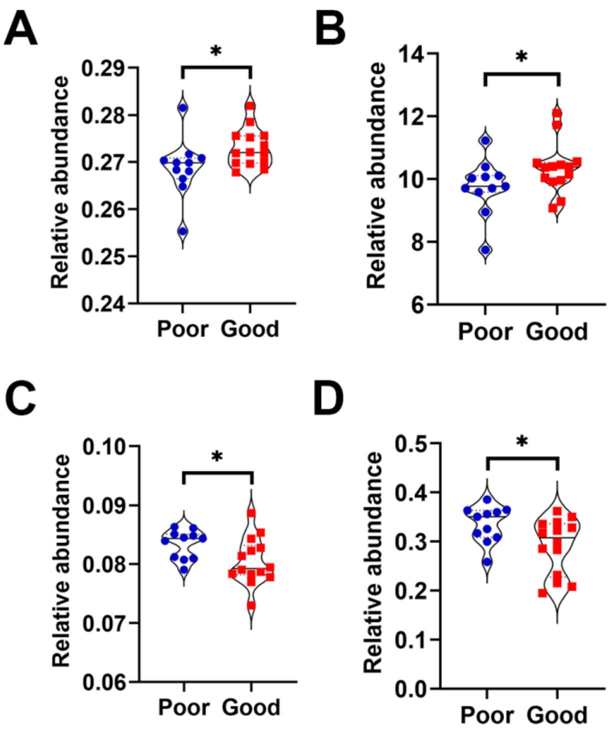

Figure S3: Examination of immune cell population in the clinical specimens. TIMER2.0 was performed on targeted-mRNA dataset to understand which cells were present in FFPE tissues from poor and good responders of anti-PD1 therapy. (A) Violin plot showing significant increase in frequencies of $CD8^+$ lymphocytes in good responders to anti-PD1 therapy estimated by TIMER. (B) Violin plot showing significant increase in cytotoxicity score of good responders to anti-PD1 therapy, estimated by MCP-counter. (C) Violin plot showing significant reduction in frequencies of $CD4^+$ lymphocytes in good responders to anti-PD1 therapy estimated by TIMER. (D) Violin plot showing significant reduction in frequencies of endothelial cells in good responders to anti-PD1 therapy estimated by EPIC. *p*-values calculated using Mann-Whitney test. $*p \geq 0.05$.
